# Propofol‐induced myoclonus during maintenance of anaesthesia

**DOI:** 10.1002/anr3.12253

**Published:** 2023-11-05

**Authors:** S. Chao, R. Khan, J. Lieberman, M. Buren

**Affiliations:** ^1^ Department of Anesthesia and Perioperative Care University of California San Francisco San Francisco California USA

**Keywords:** myoclonus, neuro‐monitoring, Propofol

## Abstract

Myoclonus is a known side effect of propofol and can interfere with surgery and possibly precipitate patient injury. Here, we report a 23‐year‐old patient undergoing an L5 osteoblastoma resection with a predominantly propofol‐based anaesthetic who developed intra‐operative myoclonus. Other adjuncts included ketamine, lidocaine and fentanyl infusions. The myoclonus did not improve after deepening the anaesthetic with propofol, opioid boluses or discontinuation of the lidocaine infusion. The myoclonus ceased after reducing the propofol infusion and increasing the ketamine and opioid infusions. The remainder of the intra‐operative course was uneventful. This report details our intra‐operative management of propofol‐induced cortical reflex myoclonus and discusses our institution's experience with treating this phenomenon.

## Introduction

Propofol, a widely used sedative‐hypnotic, is a short‐acting gamma‐aminobutyric acid receptor (GABA_A_) agonist that can safely be used as a general anaesthetic during trans‐cranial motor‐evoked potential neuromonitoring. The drug is generally well tolerated with side effects including cardiorespiratory depression and pain on injection that are easily managed in an anaesthetic context [1]. A less common side effect is seizure‐like phenomena, including myoclonus. These manifestations of neuro‐excitation are usually witnessed during the initial administration of propofol and are rarely seen during the maintenance phase of general anaesthesia [1]. Here, we report a case of propofol‐induced myoclonus during maintenance of general anaesthesia.

## Report

A 23‐year‐old man weighing 66 kg underwent an L4‐S1 posterior spinal fusion with a partial corpectomy at L5 for resection of an L5 osteoblastoma under general anaesthesia. On the morning of surgery, the patient appeared to be anxious and complained of lower back pain associated with bilateral radiculopathy. He was otherwise healthy with no allergies and no known personal or family history of neurological disorders. The patient was taking meloxicam for pain but denied using any psychotropic or antiepileptic medications. He had never undergone general anaesthesia. Other than postoperative nausea and vomiting, he had no family history of anaesthetic complications. His airway, cardiopulmonary and neurological examinations were unremarkable, and his pre‐operative blood tests were within normal limits.

The patient was pre‐medicated with paracetamol 1000 mg and midazolam 2 mg. Standard monitors were applied. The patient received an intravenous induction of general anaesthesia with fentanyl (50 μg), lidocaine (100 mg), propofol (300 mg in divided doses) and rocuronium (50 mg), and a tracheal tube was placed. An arterial line and additional peripheral intravenous cannulae were sited. A Sedline Brain Function Monitor (Masimo, Inc. Irvine, California, USA) was applied for monitoring depth of anaesthesia, and the neurophysiologist placed leads for motor‐evoked (MEP), somatosensory‐evoked potentials (SSEP), and electromyography (EMG). The SSEP leads also allow interpretation of cortical EEG activity. To facilitate neuromonitoring, anaesthesia was maintained using a total intravenous technique consisting of propofol 135 μg.kg^−1^.min^−1^, lidocaine 2 mg.kg^−1^.hr^−1^, magnesium 6 mg.kg^−1^.hr^−1^ following a 30 mg.kg^−1^ bolus over 30 min, ketamine 3 mcg.kg^−1^.min^−1^, and intermittent boluses of fentanyl. The arterial blood pressure was maintained within 20% of preoperative baseline using intravenous fluid and vasoactive agents. The patient was mechanically ventilated to maintain normocarbia and oxygen saturation was greater than 95% throughout the case. Intra‐operative warming devices were used to maintain core body temperature between 36 and 37°C.

Approximately 90 min after the start of surgery, the patient developed recurrent myoclonic jerks in his bilateral upper extremities. Each bout of myoclonus lasted for 5–10 s before spontaneously resolving and then recurring a few minutes later. These episodes did not correlate with any surgical or MEP stimulus. During these episodes, the patient remained haemodynamically stable and normothermic. Electroencephalography (EEG) showed slow‐delta and alpha oscillations consistent with an appropriate depth of anaesthesia and there was no evidence of seizure on the EEG as interpreted by the in‐room neurophysiologist. Furthermore, no lower extremity EMG activity correlated with the upper extremity myoclonic movements. This pattern of recurrent myoclonus continued for approximately 30 min. We attempted to abolish the myoclonus over the next 30 min by deepening the anaesthetic with boluses of propofol and fentanyl and increasing the propofol infusion rate with no effect. Due to concern regarding possible local anaesthetic systemic toxicity, the lidocaine infusion was discontinued at the same time. The ketamine infusion was briefly discontinued without any improvement. After consulting multiple anaesthetists, we suspected that propofol was the likely culprit and decreased the infusion to 50 mcg.kg^−1^.min^−1^. To compensate for the dose decrease, we added a small amount of sevoflurane (0.3 minimum alveolar concentration) and significantly increased the ketamine infusion to 10 μg.kg^−1^.min^−1^. To ensure amnesia and an adequate depth of anaesthesia, we also administered a 2‐mg bolus of midazolam and a 2‐mg bolus of hydromorphone. After these interventions, the myoclonic activity terminated within a few minutes and did not recur for the duration of the surgery. The patient's trachea was successfully extubated at the end of the case and recovery in the post‐anaesthesia care unit was without incident. The patient denied experiencing any complications from anaesthesia including intraoperative awareness, unpleasant post‐operative sensory perceptions or post‐operative seizures.

## Discussion

Propofol is a GABA_A_ receptor agonist that is commonly used to induce and maintain anaesthesia because of its rapid onset and offset. Its most common side effects include pain on injection and dose‐dependent cardiorespiratory depression [1]. Another known, but rare, side effect of propofol is neuro‐excitation manifesting as seizure‐like phenomena such as myoclonus or generalised tonic–clonic seizures. Case reports describing this side effect have been documented as early as the 1980s [2]. A systematic review by Walder et al. demonstrated that propofol‐induced seizure‐like phenomena is most common during induction, emergence or the immediate post‐operative period [1]. To date, there are very few reported cases of propofol‐induced seizure‐like phenomena during the maintenance phase of anaesthesia in the medcial literature. One case report described an instance of propofol‐induced status epilepticus that was refractory to benzodiazepines, antiepileptics and barbiturates but abated after discontinuing the propofol infusion [3]. This differs from our case as our patient's clinical presentation was more consistent with myoclonus, although a focal seizure cannot be ruled out from the monitoring that was available.

The mechanism of propofol‐induced neuroexcitation is unclear. Some have postulated that patients are more prone to seizure‐like phenomena whenever large changes of propofol levels in the brain occur (i.e. induction, emergence) [1]. However, our institution has seen intraoperative myoclonic movements during the maintenance phase of an anaesthetic several times, during which a steady state of propofol has been achieved. We hypothesise that the mechanism is cortical reflex myoclonus. Cortical reflex myoclonus is described as focal, affecting distal muscles, and can be triggered by various stimuli [4]. In our experience, propofol‐induced myoclonus is most common in children undergoing correction for neuromuscular scoliosis. In adults, it can occur in patients with pre‐existing grade 3 or 4 hyper‐reflexia secondary to brain or spinal cord pathology. The myoclonus is typically preceded by some kind of stimulus, often electrical cautery stimulation, and tends to develop approximately 60–90 min after surgical incision. The distribution of myoclonus is variable and can be confined to the upper or lower extremities or involve the whole body in various patterns.

Given the paucity of cases, the appropriate management of propofol‐induced seizure‐like phenomena during maintenance of anaesthesia remains unclear. In our institution, we have observed that myoclonus is successfully treated ~80% of the time by increasing the propofol infusion rate. This approach tends to be less successful in children who may require extremely high infusion rates of 250–300 μg.kg^−1^.min^−1^. Rarely, as in the case of our patient presented here, the myoclonus is resistant to higher levels of propofol, and various alternative anaesthetic techniques need to be employed. Here, we were able to suppress the myoclonus and maintain a general anaesthetic compatible with MEP and SSEP neuromonitoring with a predominantly ketamine‐based anaesthetic supplemented with sevoflurane, lidocaine and a low infusion rate of propofol.

In conclusion, propofol can in rare instances lead to neuro‐excitation events, such as myoclonus, even during the maintenance phase of anaesthesia. This underscores the necessity for vigilant monitoring and the need for a flexible anaesthetic plan. While increasing the propofol infusion rate can sometimes address this phenomenon, alternative anaesthetic techniques may be warranted. In our case, the combination of ketamine with sevoflurane and a reduced rate of propofol proved effective, highlighting the importance of individualised patient care and adaptability in anaesthetic management.
